# The Effect of Tumor Necrosis Factor-α and Interleu-Kin-1β on the Restorative Properties of Human Oligodendrocyte Precursor Cells In Vitro

**DOI:** 10.3390/bioengineering12050457

**Published:** 2025-04-25

**Authors:** Zhaoyan Wang, Ying He, Qian Wang, Weipeng Liu, Yinxiang Yang, Haipeng Zhou, Xuexia Ma, Caiyan Hu, Zuo Luan, Suqing Qu

**Affiliations:** Department of Pediatrics, The Sixth Medical Center of PLA General Hospital, Beijing 100048, China; zhaoyan_wang0527@163.com (Z.W.); jiyingbengchan1982@163.com (Y.H.); qiangcaob0955@163.com (Q.W.); choupengni1151@21cn.com (W.L.); xianguanf488@21cn.com (Y.Y.); poshai064@126.com (H.Z.); xiawen01muzhi@126.com (X.M.); zhucaiyong280@yeah.net (C.H.)

**Keywords:** premature white matter injury, TNF-α, IL-1β, human oligodendrocyte precursor cell, migration, proliferation, differentiation

## Abstract

Premature white matter injury (PWMI) represents the principal form of brain injury in preterm infants, and effective therapies remain elusive. Transplantation of oligodendrocyte precursor cells (OPCs) emerges as a potential treatment for PWMI, yet the injury-induced inflammatory response may impact these cells’ functionality. To date, no studies have explored the influence of inflammatory factors on the functionality of human (h) OPCs. The predominant inflammatory cytokines identified in PWMI lesions are tumor necrosis factor (TNF)-α and interleukin (IL)-1β. This study investigates the impact of these cytokines on hOPC migration, proliferation, and differentiation using the human adult neural stem cell amplification and differentiation system in vitro. Results indicate that IL-1β significantly impedes hOPC migration, while both TNF-α and IL-1β hinder proliferation and differentiation. In summary, inflammatory factors overexpressed following PWMI impede OPCs from realizing their regenerative potential. These findings underscore the necessity of modulating the post-PWMI inflammatory milieu to enhance the efficacy of transplanted cells concerning migration, proliferation, and differentiation.

## 1. Introduction

Preterm birth constitutes a significant global health challenge, particularly in neonatal healthcare [1]. Despite a 59% reduction in global mortality for children under five since 1990, survivors of preterm birth face an increased risk of both short-term and long-term health complications [2]. A primary concern in this demographic is Premature White Matter Injury (PWMI), a leading type of brain damage in preterm infants caused by their heightened susceptibility to factors such as hypoxia–ischemia, infection, and inflammation. PWMI notably hinders the normal development of myelin, contributing to increased neonatal mortality and chronic conditions including cerebral palsy, visual and intellectual impairments, epilepsy, and learning disabilities [3,4]. Unfortunately, there is currently no effective treatment for white matter damage in premature infants.

In recent years, cell replacement therapy has emerged as a vital and promising approach for treating central nervous system (CNS) diseases, including neurodegenerative and demyelinating disorders [5]. Oligodendrocyte precursor cells (OPCs), characterized by their ability to migrate, proliferate, and differentiate, are found throughout the CNS [6]. These OPCs evolve into myelinating oligodendrocytes (OLs), crucial for normal brain myelination, providing nutritional support to neurons and facilitating saltatory conduction of nerve impulses in the CNS [7]. Significantly, OPCs are the primary cells affected by PWMI [8]. Consequently, the transplantation of OPCs is increasingly viewed as a potential therapeutic intervention for white matter injuries [9,10].

PWMI is a complex condition characterized by a potent inflammatory cascade that includes axonal swelling, microglial cell activation, astrocytosis, and OL injury [4,11]. Research indicates that OPC transplantation may foster remyelination and restore neural function in recipients. However, the success of transplanted human OPCs (hOPCs) is contingent upon environmental conditions within the injured brain and the presence of inflammatory cytokines, which may impede the cells’ function. This potential impact remains underreported. Post brain injury, the inflammatory milieu is a critical factor limiting transplanted cells’ functionality. For instance, in cerebral leukodystrophy, inflammatory and toxic cytokines led to low survival rates of transplanted mouse neural stem cells (NSCs) and OPCs, hampering cell migration and preventing remyelination [12,13]. Similarly, Giannakopoulou et al. found that inflammatory elements in a multiple sclerosis animal model hindered the differentiation of transplanted mouse NSCs into myelinating cells [14]. Furthermore, in spinal cord injuries, this inflammatory environment reduced the survival and differentiation of transplanted NSCs [15,16]. These findings underscore the significant impact of inflammation on the functionality of transplanted human cells. Therefore, a primary objective in enhancing hOPC transplantation efficacy involves understanding how inflammatory cytokines influence hOPCs and devising strategies to manage or augment their effects.

## 2. Materials and Methods

### 2.1. hOPC Culture and Differentiation

Human neural stem cells (hNSCs) were induced to differentiate into hOPCs and subsequently into OLs, using methods previously described [17]. The hNSCs were sourced from the Department of Pediatrics of the Sixth Medical Center of PLA General Hospital. These cells were cultured in suspension for 14 days, then dissociated into single cells using 0.025% trypsin solution for 15 min at 37 °C. The digestion process was halted by adding DMEM/F-12 (Thermo Fisher Scientific, Waltham, MA, USA). Following centrifugation, the cells were seeded in six-well plates pre-coated with human fibronectin (Thermo Fisher Scientific) at a density of 2 × 104 cells/cm2 in hOPC maintenance medium, as previously detailed [17]. The medium was replenished with fresh hOPC medium every three to four days, replacing half of the medium each time. For the differentiation of hOPCs into mature OLs, the cells were treated with accutase™ cell detachment solution (BD Biosciences, San Diego, CA, USA) upon reaching 80–90% confluence. Subsequently, they were plated at a density of 1 × 104 cells/cm2 in OPC differentiation medium (OPCDM, ScienCell, Carlsbad, CA, USA) and incubated in a 5% CO_2_ environment at 37 °C. Half of the medium was refreshed biweekly. All experiments involving human neural stem cells were conducted following approval from the Ethics Committee of the Sixth Medical Center, PLA General Hospital (Approval No. [2017-SCXK-0001]). All procedures were performed in accordance with international ethical guidelines and regulatory requirements.

### 2.2. Immunocytochemistry

Monolayer cell cultures underwent fixation using 4% paraformaldehyde for 10 min. This was followed by three washes with phosphate-buffered saline (PBS). The cells were then blocked in 5% bovine serum albumin (BSA) in PBS for 2 h at 37 °C. Subsequent staining was performed overnight at 4 °C with anti-platelet-derived growth factor α (PDGFR-α) antibody (Abcam, 1:800) and anti-proteolipid protein 1 (PLP1) antibody (Abcam, Cambridge, MA, USA, 1:100) in blocking buffer. Primary antibodies were identified using suitable secondary antibodies: donkey anti-rabbit Alexa Fluor 594 (Abcam, 1:500) and donkey anti-mouse Alexa Fluor 594 (Abcam, 1:500). For the negative control, secondary antibodies were used without prior primary antibody treatment [18]. Cell nuclei were counterstained with 4′,6-diamidino-2-phenylindole (DAPI) (Beyotime, Shanghai, China). Immunostaining results were analyzed using an Olympus BX60 microscope (Tokyo, Japan).

### 2.3. Flow Cytometry

HOPCs were cultured for 7 days, followed by digestion into single cells for flow cytometry analysis. These cells were enumerated, centrifuged at 200× *g* for 5 min at 4 °C, and then blocked with Fc Block (BD Biosciences) in staining buffer (BD Biosciences) for 10 min at 25 °C. Prior to incubation with antibodies, cells were chilled on ice for 30 min. The conjugated antibodies used were BV421-platelet-derived growth factor receptor PDGFR-α (BD Biosciences), PE-A2B5 (Miltenyi Biotec, Bergisch Gladbach, Germany), and AF488-Sox10 (Novus Biologicals, Littleton, CO, USA). For non-conjugated antibodies, cells were treated with Olig2 (EMD Millipore, Billerica, MA, USA) and Alexa-488 conjugated donkey anti-rabbit (Abcam, 1:500) for 1 h. Subsequently, cells were resuspended in staining buffer, passed through a 40 µm cell strainer, and analyzed using a flow cytometer (BD Biosciences). A ‘cell’ gate, established on a forward scatter vs. side scatter plot from the isotype control group using Flow Jo 7.6 software, excluded debris. Histogram plots were generated from cell subsets, extending the gate to the far right of the histogram, defined as less than 0.5% of positive events. The proportion of positive cell populations was statistically analyzed based on histogram profiling using Overton subtraction [19,20].

### 2.4. Cell Counting Kit-8

Cell viability was evaluated utilizing the Cell Counting Kit-8 (CCK-8; Dojindo, Kumamoto, Japan), adhering to the manufacturer’s protocol. In brief, a density of 2 × 104 cells/well was cultured in 96-well plates using hOPC medium, with varying concentrations of cytokines. A cytokine-free medium served as control. The CCK-8 reagent was applied daily in a 1:10 dilution and incubated for 4 h. Optical density measurements at 450 nm were conducted using a microplate reader (BioTek, Winooski, VT, USA). This experiment was replicated thrice.

### 2.5. Migration Assay

Cell migration was assessed using a Transwell insert featuring an 8 μm pore size (Corning, Corning, NY, USA). Human oligodendrocyte progenitor cells (hOPCs) were seeded at a density of 2 × 104 cells/well in the insert’s upper chambers. These chambers contained hOPC medium supplemented with IL-1β (30, 60, 90 ng/mL) or a control vehicle, while the lower chambers held only the hOPC medium. The hOPCs were allowed to migrate for 18 h in a 5% CO_2_ environment at 37 °C. Post migration, cells remaining on the upper surface of the Transwell membrane were removed using a cotton swab. The cells that migrated to the membrane’s underside were fixed with 4% paraformaldehyde for 10 min and subsequently stained with DAPI in a 1:10 ratio for 10 min. The entire immunostained membrane was imaged using an Olympus microscope (Tokyo, Japan). The total number of migrated cells was quantified and compared to the initial number of seeded cells. Each experimental condition was replicated three times, and the results are presented as average values relative to the controls.

### 2.6. Cell Proliferation Assay

The cellular proliferation capacity was assessed through Ki-67 immunofluorescence staining. hOPCs underwent incubation with 10 ng/mL TNF-α or 30 ng/mL IL-1β for 7 days. Subsequently, the cells were blocked using a solution of 1% BSA and 0.1% Triton X-100 in PBS for one hour at 37 °C. The cells were then stained overnight at 4 °C with an anti-Ki-67 antibody (Abcam, 1:250) in the blocking buffer. Following this, the cells were washed thrice with PBS, and primary antibodies were detected using donkey anti-rabbit Alexa Fluor 488 (Abcam, 1:500). Additionally, cell nuclei were counterstained with DAPI. An Olympus BX60 microscope (Tokyo, Japan), equipped with a 20× objective lens, was used to examine the immunostaining results. The proportion of Ki-67-positive cells in the DAPI-positive population was calculated and presented as a percentage. Each experiment was independently replicated a minimum of three times.

### 2.7. RNA-Sequencing Analysis

To study the influence of TNF-α and IL-1β on the differentiation of hOPCs, compared with the control group, purified hOPCs were exposed to 10 ng/mL TNF-α or 30 ng/mL IL-1β in OPC differentiation medium for 72 h. RNAiso Plus (Takara Bio, Kusatsu, Japan) was used for isolating the total RNA of the three samples. RNA-seq experiments were performed using Novogene (Beijing, China). A NanoPhotometer^®^ spectrophotometer (Implen, Westlake Village, CA, USA) was used to assess RNA purity. An RNA Nano 6000 Assay Kit with the Bioanalyzer 2100 system (Agilent Technologies, Santa Clara, CA, USA) analyzed RNA integrity. According to manufacturer’s instructions, the sequencing library was generated using the Illumina (San Diego, CA, USA) NEBNext Ultra™ RNA Library preparation kit. The sequence of each sample has a corresponding index code. The TruSeq PE Cluster Kit v3-cBot-HS (Il-lumina) was used to cluster the index-coded samples on a cBot Cluster Generation System. The Illumina NovaSeq platform was used to prepare the sequencing library to generate 150 bp paired-end reads. We performed normalization and differential expression analysis using the DESeq2 package (v1.30.0). Genes with an adjusted *p*-value (padj or FDR) < 0.05 and |log2FoldChange| ≥ 1 were considered differentially expressed genes (DEGs). Differentially expressed genes were analyzed for gene ontology (GO) enrichment through the cluster Profiler R package. We included three biological replicates per condition (Control, TNF-α, IL-1β) to ensure the reliability of our results. After performing differential expression analysis, we conducted gene set enrichment analysis (GSEA) to evaluate the functional pathways potentially modulated by inflammatory factors. All genes were ranked by their log2FoldChange in descending order, and GSEA was carried out in the R environment using a commonly used package for pathway enrichment. The gene sets were mainly derived from public repositories such as MSigDB, with predefined minimum and maximum gene set sizes. We employed permutation-based methods to assess the statistical significance of enrichment, and a false discovery rate (FDR) threshold of 0.05 was used to define significantly enriched pathways. This allowed us to pinpoint key biological processes that might be influenced by TNF-α or IL-1β, thereby providing deeper insight into how these inflammatory factors affect hOPC differentiation.

### 2.8. Statistical Analyses

Student’s *t*-test and one-way analysis of variance (ANOVA) were employed to compare means between two groups and among multiple groups, respectively. For multiple comparisons, Dunnett’s post hoc test was applied. Data are presented as means ± standard deviation (SD). A *p*-value of 0.05 or less was considered statistically significant.

## 3. Results

### 3.1. OLs Are Efficiently Established from hNSC-Derived hOPCs

**Figure 1:**
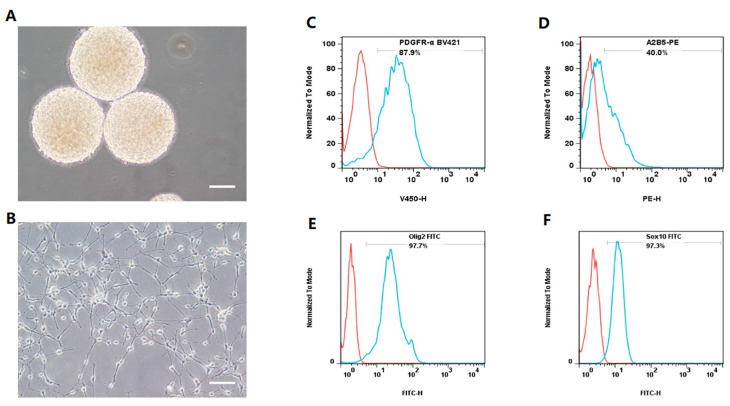

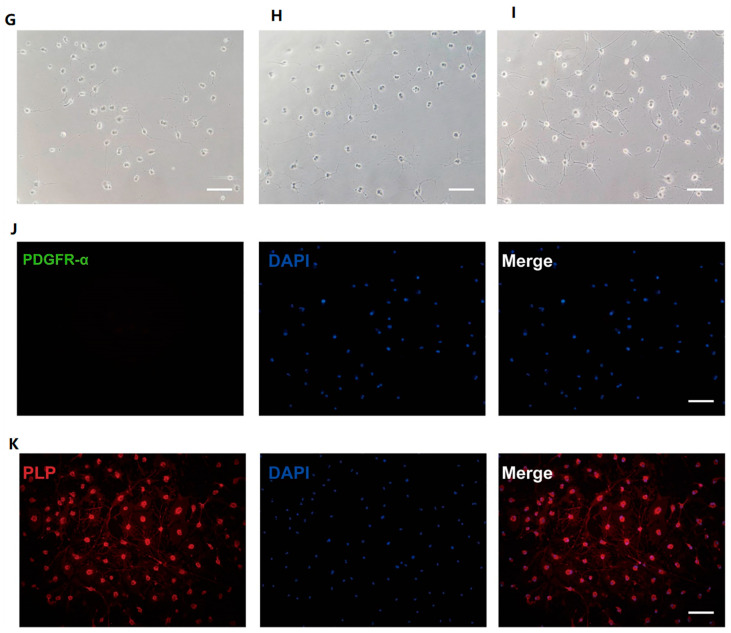
Induction of human oligodendrocyte precursor cells (hOPCs) and oligodendrocytes (OLs) derived from human neural stem cells (hNSCs). (**A**) hNSCs were in suspension culture. (**B**) hOPCs were cultured adherently for 7 days. (**C**–**F**) Representative flow cytometry plots of specific markers within the hOPCs population: (**C**) PDGFR-α, (**D**) A2B5, (**E**) Oig2, and (**F**) Sox10. The morphology of OLs is changed at (**G**) 3, (**H**) 9, and (**I**) 16 days. Mature OLs do not express PDGFR-α (**J**) proteolipid protein 1 (PLP1) (**K**). Scale bars, 100 μm. The red outline represents negative cells, and the blue outline represents positive cells (**C**,**D**,**E**,**F**).

hNSCs were maintained in suspension culture, forming neurospheres measuring 200–250 μm in diameter (Figure 1A). Subsequently, these hNSCs were digested and directly differentiated into hOPCs. After a week of culture, there was a significant increase in the number of hOPCs exhibiting typical bipolar or tripolar processes, indicative of immature cells (Figure 1B). Flow cytometry analysis revealed that 89.3 ± 2.13%, 40.1 ± 4.36%, 98.1 ± 1.3%, and 95.2 ± 2.23% of the cells expressed PDGFR-α (Figure 1C), A2B5 (Figure 1D), Olig2 (Figure 1E), and Sox10 (Figure 1F), respectively. These high expression rates of specific markers confirmed the successful induction of a high-purity hOPC population from hNSCs. The hOPCs were then differentiated into mature OLs. Three days into the differentiation process, the cells exhibited multiple branching processes (Figure 1G). By day 9, they developed a complex multipolar morphology with numerous small branches (Figure 1H). After 16 days, the differentiated cells displayed multipolar and web-like processes or membrane-like structures and were subject to immunofluorescence staining (Figure 1I). The differentiated cells lacked PDGFR-α expression (Figure 1J) but were positive for PLP1 (Figure 1K), displaying web-like and membrane structures, confirming their maturation into OLs.

### 3.2. TNF-α and IL-1β Inhibit hOPC Migration

**Figure 2:**
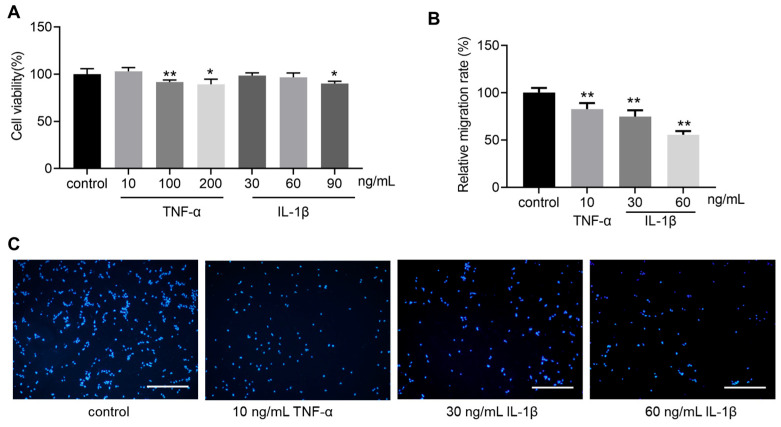
Effect of inflammatory cytokines on human oligodendrocyte precursor cell (hOPC) migration. (**A**) CCK-8 assay showing the effect of various concentrations of TNF-α (10, 100, 200 ng/mL) or IL-1β (30, 60, 90 ng/mL) on hOPC viability after 18 h. (**B**) Relative migration rate of hOPCs under TNF-α or IL-1β treatment compared to the control group. (**C**) Representative DAPI-stained images of hOPCs that migrated through the Transwell membrane. Scale bar = 200 µm. Data are expressed as mean ± SD. * *p* < 0.05 vs. control group; ** *p* < 0.01 vs. control group.

The initial step in remyelination involves the migration of activated OPCs to the site of damage. To assess the impact of inflammatory factors on hOPCs migration, a Transwell assay was conducted. To negate the potential cytotoxic effects of TNF-α or IL-1β on hOPCs, cells were incubated with TNF-α (10, 100, 200 ng/mL) or IL-1β (30, 60, 90 ng/mL), without employing Transwell. Following 18 h of culture, as shown in Figure 2A, although higher concentrations (e.g., 200 ng/mL TNF-α and 90 ng/mL IL-1β) led to a moderate decrease in cell viability, the lower concentrations did not significantly affect hOPCs viability within this short-term exposure. Next, we employed a Transwell assay to assess how these inflammatory factors influence hOPC migration (Figure 2B). hOPCs were seeded in the upper chamber with medium containing TNF-α or IL-1β, while the lower chamber contained cytokine-free medium. After 18 h, cells that migrated to the underside of the Transwell membrane were fixed and stained with DAPI. We observed that 10 ng/mL TNF-α or 30/60 ng/mL IL-1β significantly reduced the number of migrated hOPCs compared to the control group, indicating a concentration-dependent inhibitory effect on hOPC migration (Figure 2B). Representative fluorescent images of migrated cells are shown in Figure 2C.

### 3.3. TNF-α and IL-1β Inhibit hOPC Proliferation

**Figure 3:**
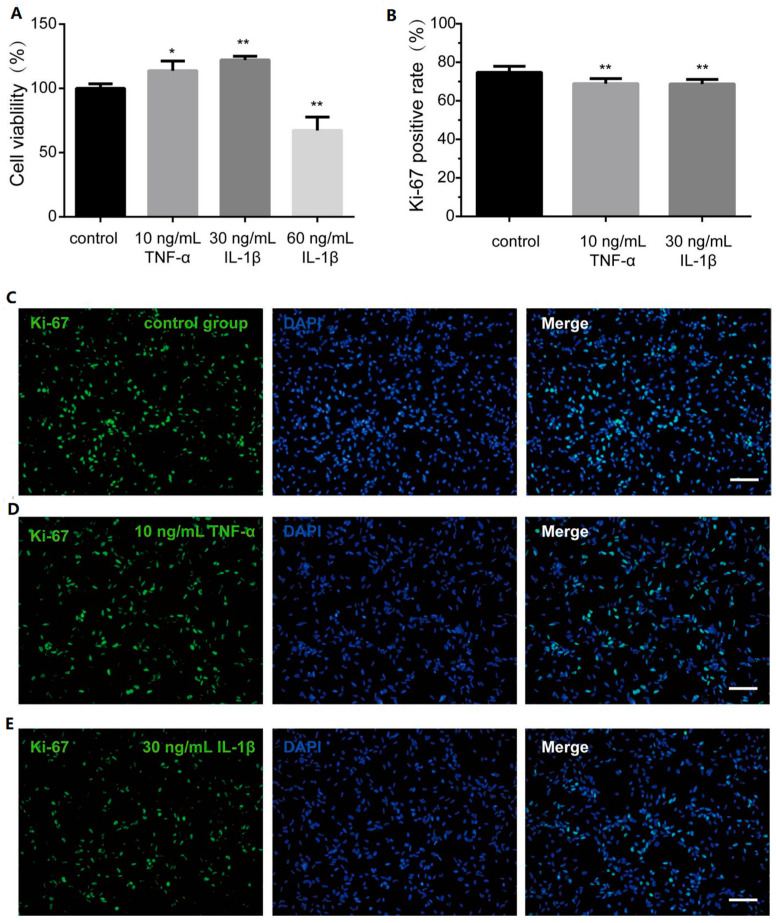
Proliferation capacity of hOPCs in the presence of inflammatory cytokines. (**A**) CCK-8 assay was performed to analyze the effect of inflammatory cytokines on cell viability. (**B**) The bar graph showed proliferation marker Ki-67 positive rate in control group and inflammatory group. The immunocytochemistry picture displayed Ki-67 in (**C**) control group, (**D**) 10 ng/mL TNF-α group, (**E**) 30 ng/mL IL-1β group. Scale bar, 100 µm. Data are expressed as the means ± SD. * *p* < 0.01 vs. control group. ** *p* < 0.001 vs. control group.

hOPCs are more likely to effectively myelinate when they proliferate adequately in areas of damage. Prior to assessing proliferation, a 7-day CCK-8 assay was conducted to evaluate cell viability in response to 10 ng/mL TNF-α, and 30 and 60 ng/mL IL-1β. Results indicated that cell viability diminished at 60 ng/mL IL-1β, whereas 10 ng/mL TNF-α and 30 ng/mL IL-1β significantly enhanced it (Figure 3A). To further investigate hOPCs’ proliferation under the influence of 10 ng/mL TNF-α and 30 ng/mL IL-1β, the proliferation marker Ki-67 was analyzed using immunocytochemistry. Ki-67 positivity revealed that the inflammatory cytokines somewhat reduced the proliferation of hOPCs after seven days (Figure 3B–E).

### 3.4. TNF-α and IL-1β Inhibit PLP1 Expression in Differentiated Cells

**Figure 4:**
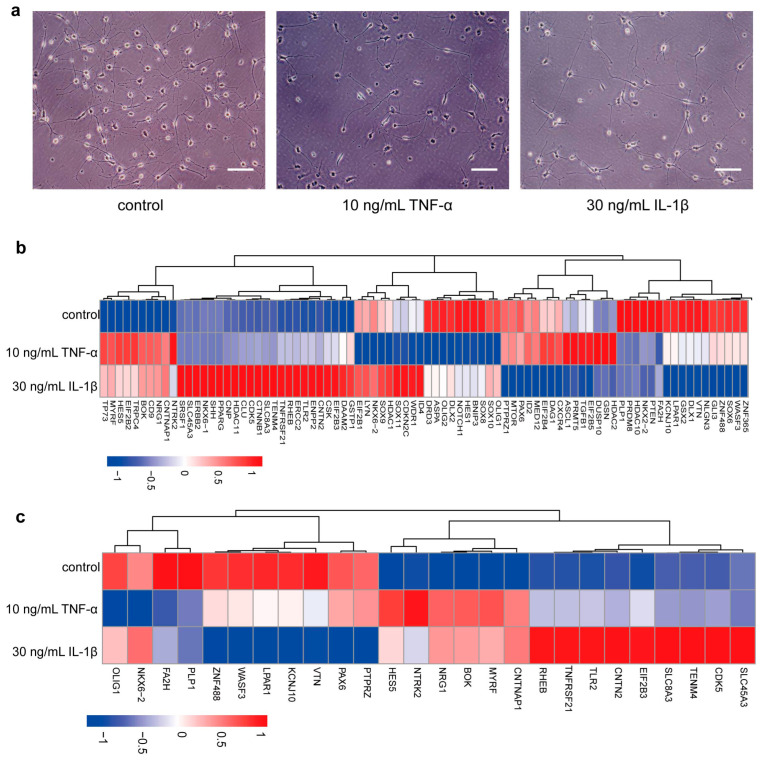
Inflammatory factors affect the morphology and gene expression of OLs derived from hOPCs. (**a**) The cell morphology is different among the cytokine-treated group and the control group. (**b**) The heatmap shows the 85 genes related to oligodendrocyte differentiation. (**c**) The heatmap shows the differentially expressed genes (DEGs) selected from 85 genes. Scale bar—100 µm.

The terminal phase of remyelination involves the maturation of OPCs into mature OLs. To explore the impact of inflammatory factors on hOPC differentiation, we administered 10 ng/mL of TNF-α or 30 ng/mL of IL-1β to the hOPC differentiation system. Subsequent analysis revealed a notable reduction in the complex branching of OLs following exposure to these cytokines (Figure 4A). The introduction of these inflammatory cytokines noticeably impeded the morphological development of the cells.

RNA sequencing (RNA-seq) analysis was conducted on OLs treated with inflammatory cytokines and compared with a control group. Focusing on the effects of these factors on differentiation, we identified 85 genes related to OL differentiation across the three groups, as depicted in the heatmap (Figure 4B). Relative to the control group, in the TNF-α treated group, seven genes showed increased expression and four exhibited decreased expression (Figure 4C). In the IL-1β-treated group, 13 genes were upregulated and 8 were downregulated (Figure 4C). Gene ontology (GO) analysis of these differentially expressed genes (DEGs) indicated significant enrichment in oligodendrocyte differentiation (GO:0048709) pathways, particularly among downregulated DEGs in both the TNF-α and IL-1β groups (*p* = 0.022 and *p* = 0.035, respectively). Notably, Olig1 and PLP1 were the primary genes affected in the TNF-α group, while PTPRZ, WASF3, KCNJ10, and PLP1 were most impacted in the IL-1β group. Although upregulated DEGs were also significantly enriched in the oligodendrocyte differentiation pathway in the IL-1β group (*p* = 0.020), TNFRSF21 was the primary gene affected.

**Figure 5:**
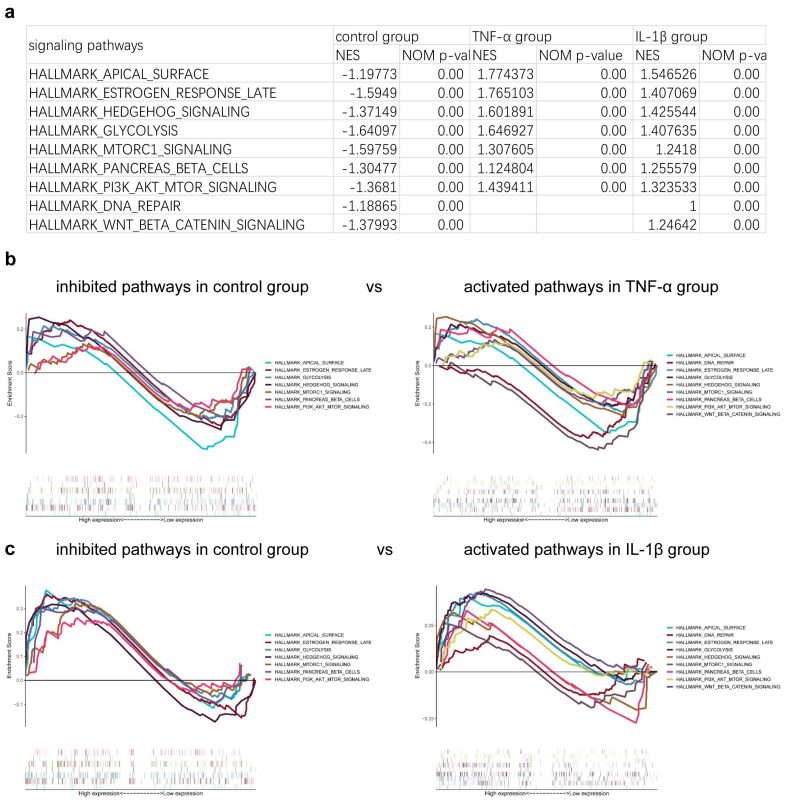
The signaling pathways correlated with differentiation were analyzed with gene set enrichment analysis (GSEA). The related signaling pathways are shown (**a**) in table and (**b**,**c**) pictures.

Gene set enrichment analysis (GSEA) was applied to cells in both the control and inflammatory groups to identify differences in activated or repressed cell differentiation signaling pathways. The results indicated that the introduction of inflammatory factors led to the activation of pathways that maintain cellular pluripotency, including those involved in DNA repair and the hedgehog signaling pathway (Figure 5a,b). This suggests that inflammatory factors may hinder the process of cell differentiation.

**Figure 6:**
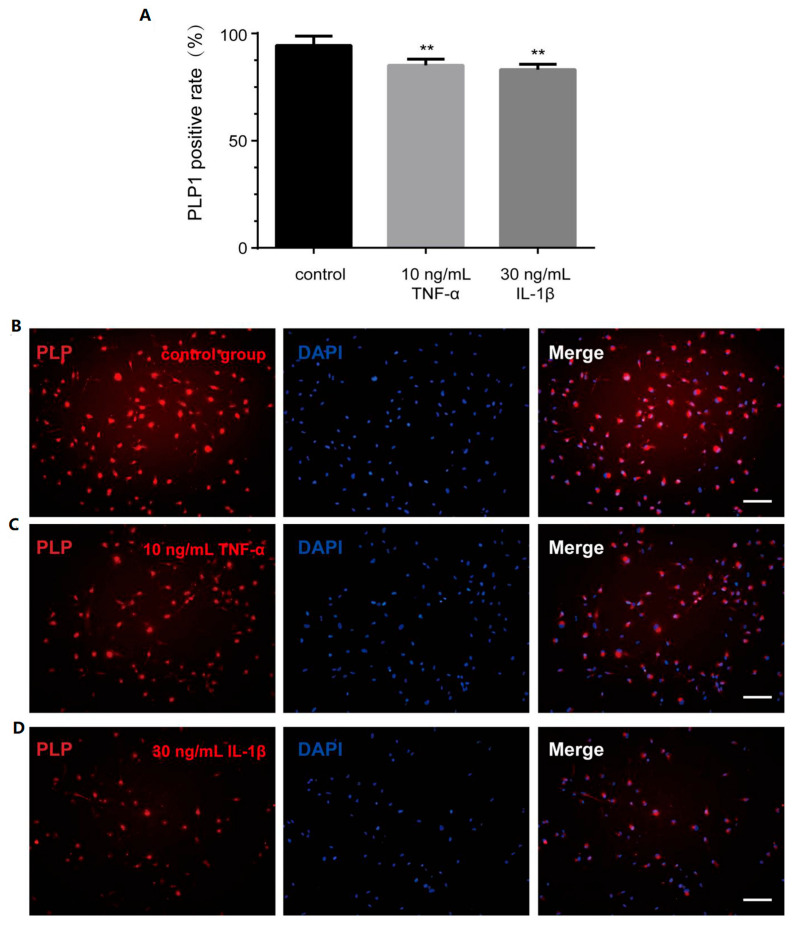
Inflammatory factors affect the PLP1 expression of OLs. (**A**) The positive rate of PLP1 in control group and inflammatory cytokines group. The immunocytochemistry picture displays PLP1 in (**B**) control group, (**C**) 10 ng/mL TNF-α group, (**D**) 30 ng/mL IL-1β group. Scale bar—100 µm. ** *p* < 0.001 vs. control group.

The expression level of proteolipid protein 1 (PLP1) is a recognized marker of cell differentiation and maturation [21]. Consequently, we assessed PLP1 expression through immunocytochemistry staining. Notably, the proportion of PLP1-positive cells decreased significantly following treatment with either TNF-α or IL-1β (Figure 6A–D).

## 4. Discussion

To find treatment for PWMI, scientists have performed many explorative studies, utilizing, for example, receptor antagonist drug, growth factor, erythropoietin, and hypothermia therapies. However, the results so far have been unsatisfactory because these methods may be unsafe and cause serious adverse reactions [3,22]. Additionally, damage to OPCs that are responsible for the differentiation into OLs in PWMI impairs myelination. Therefore, OPC transplantation is a potentially effective method for treating PWMI. Indeed, scientists have found that multiple sources of OPCs are effective against periventricular leukomalacia, improving neurobehavior and providing neuroprotective effects [23,24,25]. However, Webber et al. found that at seven weeks after transplantation in periventricular leukomalacia, OPCs survived and differentiated into an immature stage in vivo but did not further differentiate into mature OLs [25]. Therefore, there remains much to be studied about the origin of OPCs and their migration into the lesion and differentiation into mature OLs.

We first investigated the origin of the OPCs. In recent years, several studies have re-ported these cells can be derived from induced-pluripotent stem cells, embryonic stem cells (ESCs), or even brain tissue [9,10,26]. However, the wide application of OPCs from ESCs or induced-pluripotent stem cells is limited by the long culture time required, scarce brain tissue sources, and the lack of safety and stability of gene modification techniques [27,28,29]. In this study, we used hOPCs derived from previously established human adult NSCs [17]. These cells expressed PDGFR-α, A2B5, Olig2, and Sox10, and differentiated into mature OLs expressing characteristic marker PLP1 instead of PDGFR-α in vitro, providing a more readily available OPC source for treating PWMI. We observed that approximately 40% of our hOPCs express A2B5, whereas PDGFR-α, Olig2, and Sox10 are detected in 90% or more of the cells. We believe this difference could be attributed to the developmental stage of the OPCs in our culture system. A2B5 expression may peak at a specific phase of OPC differentiation and then decline at other stages. Thus, not all cells in our culture may be at the exact stage where A2B5 is highly expressed.

It is considered that, at the peak of PWMI (23–32 weeks of gestation), OL lineages, including OPCs and pre-OLs, are more vulnerable to hypoxic and ischemic injury [3,30]. We hypothesized that OPCs are also susceptible to the inflammatory environment after PWMI, which is one of the reasons behind hOPC transplantation for PWMI. Therefore, studying the effect of the inflammatory environment after PWMI on OPC function is helpful to understand the fate and improve the efficacy of hOPCs after transplantation. Studies have shown that hypoxia–ischemia stimulates inflammatory responses, producing high levels of TNF-α and IL-1β in PWMI diseases [11]. Migration to the lesion, followed by proliferation and differentiation after transplantation, are important factors affecting the efficacy of OPC transplantation. Based on a previously established culture system for hOPC amplification and differentiation into OLs, we focused on the influence of TNF-α and IL-1β on OPC migration and proliferation and OL differentiation.

OPC migration is the first step for achieving effective transplantable cell therapy. In our experiment, CCK-8 assay confirmed that 30 or 60 ng/mL IL-1β had no cytotoxicity on hOPCs. However, 90 ng/mL IL-1β had a cytotoxic effect on hOPCs, which may cause cell death and reduce the living cell number. Vela et al. showed that IL-1β differed in its effect from other pro-inflammatory cytokines or stress-inducing factors and that 5–60 ng/mL IL-1β had no toxic effect on oligodendrocyte lineage cells and could not activate the signaling pathway of OPC death [31], which is in agreement with our results. In Transwell assay, IL-1β (30 or 60 ng/mL) significantly inhibited hOPC migration in a concentration-dependent manner. The in vitro migration results of IL-1β were consistent with those of in vivo studies [32], and those authors suggested that the most important reason for the disruption of myelin regeneration in chronic cerebral hypoperfusion was that IL-1β inhibited the migration of OPCs from the subventricular region to the corpus callosum [32]. Thus, 30 or 60 ng/mL IL-1β, rather than the cytotoxicity induced by inflammatory cytokines, directly reduced the number of migrated cells. During normal development or inflammatory cytokines stimulation in rodent animals, OPCs expressing migratory receptors such CXCR4 or CXCR2 migrated to white matter or lesion areas [33,34]. Future experiment may focus on the expression of such receptors in hOPCs stimulated by inflammatory cytokines. The migratory inhibition by 60 ng/mL IL-1β, which was more obvious than that by 30 ng/mL IL-1β, and the cytotoxicity of 90 ng/mL IL-1β suggested that transplantation performed at the peak concentrations of the cytokines may be detrimental to the primary ability of cells to migrate. Therefore, in addition to hOPC transplantation, the level of inflammatory factors in vivo should be regulated to improve the cell migration potential.

In the present study, we employed a conventional Transwell assay to evaluate how inflammatory cytokines influence the migration of hOPCs. While this method provides a straightforward measure of the number of cells crossing from the upper to the lower chamber, it does not differentiate between various mechanisms that may reduce hOPC migration. For instance, an observed decrease in cells at the bottom chamber could result from direct inhibition of motility, or from chemotactic/attractive signals that encourage cells to remain in the upper chamber. Placing IL-1β in the lower chamber might have offered additional insights, but would still pose challenges in distinguishing repulsion from reduced motility. Moreover, Transwell assays do not typically capture dynamic changes or allow real-time observation of cell movement. Alternative approaches, such as microfluidic gradient systems or time-lapse imaging, can more precisely dissect whether IL-1β exerts a chemotactic, repulsive, or purely inhibitory effect on OPCs. Nevertheless, our primary aim was to assess the overall impact of inflammatory factors on hOPC migration rather than to pinpoint specific chemotactic mechanisms. Future studies focusing on microenvironmental cues, receptor expression (e.g., CXCR2, CXCR4), and direct imaging will help clarify whether IL-1β exerts its effects through attraction, repulsion, or other pathways.

One of the reasons for the failure of transplantation is the small number of cells. In addition to a sufficient number of cells originally, the transplanted cells must have a strong ability to proliferate, reaching enough cell number to differentiate and improve the possibility of remyelination. OPCs are a highly proliferative cell population, which play an important role in the efficacy of transplantation. The CCK-8 kit showed that hOPCs viability decreased stimulated with 60 ng/mL IL-1β. Therefore, the high concentration of inflammatory environment has a toxic effect on cells survival, and it is difficult for cells to proliferate to the appropriate number and achieve curative repairment effect. However, 10 ng/mL TNF-α and 30 ng/mL IL-1β had no toxic effect on the cells. The proliferation of cells was evaluated by immunofluorescence staining with the proliferation marker Ki-67. Without the stimulation of inflammatory factors, human OPCs have a higher proliferation ability, which is conducive to reach a large number of cells. However, the persistence of inflammatory factors reduced the positive rate of Ki-67 in hOPCs, suggesting that inflammatory factors inhibited the proliferation of hOPCs. It has been reported that TNF-α and IL-1β can inhibit the proliferation of rat OPCs in vitro [35,36]. It can be inferred from these in vitro results that despite the successful localization of OPCs to the lesion site, the persistent inflammatory factors are still inhibiting the proliferation effect of transplanted cells. In this study, we did not examine the effects of TNF-α or IL-1β on other hOPC markers such as PDGFR-α, A2B5, Olig2, and Sox10. The expression changes in these markers would be essential for a comprehensive understanding of how inflammatory cytokines influence hOPC maturation. Therefore, we plan to verify them in our future research using flow cytometry, immunofluorescence, or Western blot, aiming to provide a more complete picture of the impact of TNF-α or IL-1β on hOPC differentiation. We believe that these forthcoming studies will fill the current gap and shed further light on potential therapeutic targets in neurological disorders.

In addition, in vivo, TNF-α binding to TNF-R2 of the host OPCs was found to promote proliferation in the demyelination model of cuprizone administration. This model involves injecting IL-1β into a traumatic brain injury model, and studies have shown that IL-1β fails to affect host OPC proliferation [37,38]. Obviously, the inflammatory response involves interaction between different cells and between cells and extracellular environ-mental factors. On the one hand, inflammatory cytokines directly damage survival, proliferation, migration, and other processes. On the other hand, cytokines rely on other cells to cause a toxic effect or a more severe inflammatory response. In order to further enrich the understanding of the efficacy of hOPC transplantation, it is necessary to further clarify the proliferation effect of hOPCs in the inflammatory environment in vivo and improve the microenvironment or enhance the function of hOPCs, promoting the development of transplantation therapy.

From the perspective of translational medicine, the transplantation of OPCs into the CNS has the important therapeutic significance of transplanted hOPCs, remyelination [39]. At present, most studies in vitro focus on the effect of inflammatory cytokines on the differentiation of OPCs instead of migration and proliferation. The current understanding of the mechanism of OPC differentiation mediated by inflammatory cytokines in vitro comes from rodent studies [35,40]. However, the effect of inflammatory cytokines on the differentiation of hOPCs and the mechanism remain to be further explored. Therefore, our experiment mainly analyzed the effects of inflammatory cytokines on the differentiation of hOPCs based on morphological and immunocytochemistry. And RNA-seq analyses were performed to find the mechanism of differentiation. The results showed that after treatment with inflammatory factors, OLs exhibited reduced branching, which was consistent with the results of a previous study [41]. RNA-seq analysis was performed to discover DEGs or new genes in the control group, 10 ng/mL TNF-α-treated group, and the 30 ng/mL IL-1β-treated group. Among the downregulated DEGs enriched in oligodendrocyte differentiation (GO: 0048709), besides PLP1, Olig1 expression also decreased in the TNF-α-treated group. In the IL-1β-treated group, except for PLP1, PTPRZ, WASF3, and KCNJ10 were significantly decreased. All of the mentioned genes were reported to correlate with OPC differentiation. Olig1 regulates the transcription of major myelination-specific genes, including PLP1, myelin basic protein (MBP) and myelin-associated glycoprotein (MAG) [42]. WASF3-dominated actin dynamic regulation is involved in the process of morphological changes in differentiated cells and axonal ensheathment [43]. Oligodendrocyte differentiation (GO: 0048709) was upregulated in the IL-1β-treated group rather than in the TNF-α-treated group. TNFRSF21 was primarily up-regulated in IL-1β-treated group. Overexpression of TNFRSF21 in OLs induced apoptosis mediated by caspases and inhibited differentiation [44]. The literature showed that the abovementioned genes are involved in differentiation through different molecular mechanisms. It provides more important bases and new ideas for understanding the molecular mechanism of CNS myelination under the stimulation of inflammatory cytokines.

Further RNA-seq analysis showed that inflammatory factors activated the related pathways of differentiated cells which keep cells in the pluripotent state such as DNA repair, hedgehog, and so on. For example, the hedgehog signaling pathway, playing a specific stage role in the regulation of OPC, was activated to stimulate cell proliferation and inhibit cell differentiation in the later period of the OPC lineage [45]. This suggests that inflammatory factors may be involved in the inhibition of cell differentiation-related pathways, and the relationship between these pathways and OPC differentiation needs to be further explored in the future.

Even though the expression of many genes associated with regulation of differentiation was altered, as shown by RNA-seq, the expression of the specific marker PLP1 was the main factor to evaluate the differentiation of OLs. It is reported that PLP1 is the most abundant intact membrane protein of myelin in the CNS, accounting for 50% of the total protein [46,47]. PLP1 plays an important role in OL maturation; interactions between OLs and axons in the early stages; membrane adhesion and compaction; and maintenance of myelin sheath stability [21,46]. Therefore, the expression of PLP1 was further determined by immunocytochemistry. The significant reduction in PLP-positive rate confirmed that inflammatory cytokines inhibited cell differentiation, which is consistent with Jana’s conclusion [48]. In terms of cell morphology, PLP1 gene expression, and positive rate, it was suggested that inflammatory cytokines after PWMI had a negative impact on the most important differentiation function of transplanted hOPCs.

## 5. Conclusions

The present in vitro studies revealed that IL-1β impedes the migration of hOPCs, while both TNF-α and IL-1β hinder hOPCs proliferation and differentiation. Despite the limitations of in vitro experiments in replicating the intricacies of PWMI in vivo, the findings of this research contribute significantly to a nuanced understanding of how inflammatory factors impact OPC functionality. This, in turn, could enhance the effectiveness of cell transplantation therapies. Consequently, it is recommended that animal studies be conducted to corroborate these findings, which are currently restricted to the cellular level.

## Data Availability

Data are contained within the article.
